# Generation of a Multicomponent Library of Disulfide Donor-Acceptor Architectures Using Dynamic Combinatorial Chemistry

**DOI:** 10.3390/ijms160716300

**Published:** 2015-07-17

**Authors:** Wojciech Drożdż, Michał Kołodziejski, Grzegorz Markiewicz, Anna Jenczak, Artur R. Stefankiewicz

**Affiliations:** 1 Faculty of Chemistry, Adam Mickiewicz University, Umultowska 89b, 61-614 Poznań, Poland; E-Mails: wojtek.dr888@gmail.com (W.D.); mic.kolodziejski1@gmail.com (M.K.); grzegorz_markiewicz@hotmail.com (G.M.); anna.jenczak@op.pl (A.J.); 2 Centre for Advanced Technologies, Adam Mickiewicz University, Umultowska 89c, 61-614 Poznań, Poland

**Keywords:** supramolecular chemistry, dynamic combinatorial chemistry, disulfides, donor-acceptor interactions

## Abstract

We describe here the generation of new donor-acceptor disulfide architectures obtained in aqueous solution at physiological pH. The application of a dynamic combinatorial chemistry approach allowed us to generate a large number of new disulfide macrocyclic architectures together with a new type of [2]catenanes consisting of four distinct components. Up to fifteen types of structurally-distinct dynamic architectures have been generated through one-pot disulfide exchange reactions between four thiol-functionalized aqueous components. The distribution of disulfide products formed was found to be strongly dependent on the structural features of the thiol components employed. This work not only constitutes a success in the synthesis of topologically- and morphologically-complex targets, but it may also open new horizons for the use of this methodology in the construction of molecular machines.

## 1. Introduction

The emerging area of molecular and supramolecular multi-component synthesis is very promising, since it becomes possible to incorporate multiple molecular building blocks in combination with multiple covalent and/or noncovalent bonds to create functional architectures with applications in, for example, recognition, sensing, catalysis, transport and encapsulation [1,2]. Despite the remarkable success of dynamic combinatorial and supramolecular chemistries, the construction of dynamic multi-component architectures or devices with interesting morphological and structural features is still challenging [3]. Nowadays, the main difficulty is to find an easy way to link together multiple distinct molecular components and to control the communication between them [4,5,6,7,8]. Thus, the development of dynamic multi-component assemblies generated through the formation of reversible covalent or noncovalent bonds is at the forefront of recent research on supramolecular chemistry, with the ultimate goals of creating molecular machines, complex functional nanoarchitectures, dynamic combinatorial libraries and sensors [9,10,11,12]. Reversible bonding (covalent and noncovalent) [13,14,15,16] is often employed for the creation of such novel molecular and supramolecular structures, multi-component assemblies and sensing ensembles [17,18,19]. Currently, the main stream of investigation is directed towards finding quick and efficient ways to control and reversibly modify the composition of molecular or supramolecular architectures [20].

The attractions between π-system moieties have long been investigated as a means of molecular recognition and self-organization [21,22,23]. Such attractions become especially useful tools for molecular recognition when originating from the strong electrostatic interactions between electron-deficient (acceptor) and electron-rich π-systems [24]. The complementary alternating stack of electron-deficient and electron-rich π-system moieties, leading to the optimum electronic overlap, has always been thought to be the most favorable arrangement. Alternating donor–acceptor (D–A) stacks adopt a parallel and compact face-to-face geometry (the latter are usually offset) and have been extensively used as scaffolds for complex structures, such as foldamers, rotaxanes, catenanes and conjugated polymers [25,26,27].

In disulfide dynamic combinatorial chemistry (DCC), it has been found that the attraction between electronically-rich and electronically-deficient aromatics can be strong enough to drive the self-assembly of many complex molecules [28,29,30,31]. In equilibrating systems, stabilization through the formation of D–A interactions is reflected in a combination of both enthalpic and entropic factors [28,32,33,34]. To construct and control supramolecular structures based on D–A systems, a more detailed understanding of the scope, limitations and geometries of the underlying D–A interactions is required. One of the most fruitful ways to uncover this is through the application of a DCC approach [9,17,35,36,37]. In DCC, building blocks are connected to each other through reversible bonds to form a pool of interconverting compounds, the dynamic combinatorial library (DCL) [38]. This reversibility means that the DCL is under thermodynamic control, the most stable species being selected by the system according to the specific conditions [39]. Using reversible chemistry introduces a degree of proof-reading into a reaction; it does not matter if the “wrong” product is formed fastest, since as long as all steps in the reaction are reversible, the system will eventually reach equilibrium, giving the most stable set of products [40]. With the use of an appropriate stimulus (chemical, physical or structural modification), the equilibrium can be shifted to produce the desired product [14].

Herein, we report a new generation of D–A disulfide architectures obtained in aqueous solution at physiological pH. The application of the DCC approach allowed us to generate a large number of new disulfide macrocyclic architectures. Furthermore, we were able to identify a new type of [2]catenane consisting of four distinct components. Up to fifteen types of structurally-distinct dynamic architectures have been generated through the one-pot disulfide exchange reaction between four thiol-functionalized aromatic components in water. The distribution of disulfide products formed was found to be strongly dependent on the structural features of the thiol components employed. This work not only constitutes a success in the synthesis of topologically- and morphologically-complex targets, but it may also open new horizons for the use of this methodology in the construction of, e.g., molecular machines.

## 2. Results and Discussion

### 2.1. Design and Structural Features of Library Components

**Figure 1:**
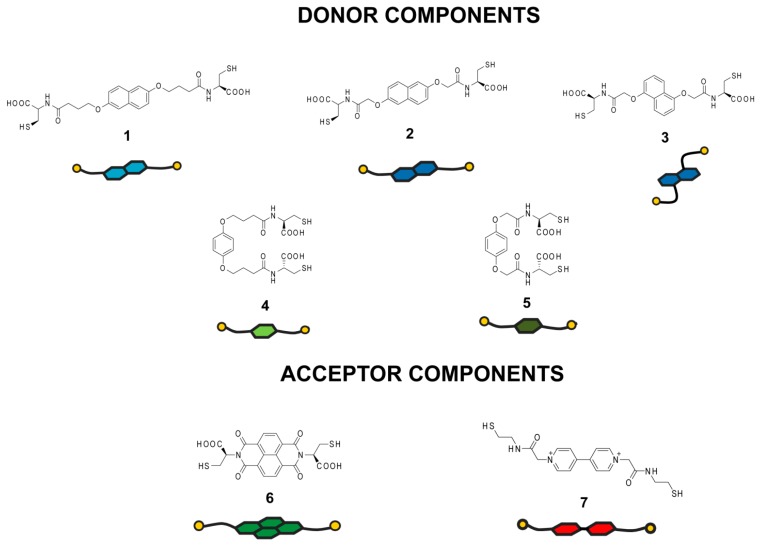
Structures and schematic representations of the thiol-functionalized components employed in the generation of a dynamic combinatorial library (DCL) in water.

To realize our strategy, a library of structurally- and electronically-distinct components has been designed and synthesized (Figure 1). All of the building blocks used in this study contain a central aromatic core with attached flexible side-chains possessing two thiol moieties. To eliminate possible differences in the rate of exchange reactions between thiol components during disulfide formation, aliphatic thiol functionalities were employed exclusively. The molecules were divided into two groups according to their electronic properties. The first group (Molecules **1**–**5**) consisted of five structures with well-established electron-donor properties. Three of them (Molecules **1**–**3**) possess electron-rich naphthalene moieties in the central part of the molecule, whereas the other two (Molecules **4** and **5**) have also an electron-rich benzene ring as a central core. While the role of the central aromatic core is to engage in D–A and/or hydrophobic interactions, attachment of two cysteine/thiol moieties provides both carboxylate anions (Molecules **1**–**6**) for water solubility at neutral pH and thiol groups for reversible covalent chemistry. In order to examine the impact of structural properties on the effectiveness of these components in the generation of complex molecular architectures in water, we introduced different lengths (the number of –CH_2_– units between aromatic core and cysteine) and positions (a 2,6- *vs.* a 1,5-substitution pattern of the naphthalene core) of the functional cysteine groups. These variations have been found to have a tremendous effect on the structural and morphological properties of disulfide systems obtained in aqueous media [30].

The second group of structures (Molecules **6** and **7**) was chosen for its ability to interact with electron-rich aromatic systems. Among the different electron-deficient units, imide-functionalized arenes are among the most important building blocks for the generation of structurally-complex molecular and supramolecular architectures, such as catenanes, rotaxanes, cages or polymers, due to their chemical accessibility, highly electron-deficient character and the solubilizing ability provided by *N*-alkylation [41]. Due to these properties, cysteine-functionalized naphthalenediimide (**6**) has been selected, synthesized and employed in our studies [23].

Viologens, formally known as 1,1′-disubstituted-4,4′-bipyridilium salts, are a well-known class of electrochromic compounds and are excellent electron acceptors [42]. The interesting physicochemical properties and good solubility of these molecules in aqueous media stimulated us to prepare a new type of thiol-functionalized Component **7** and investigate its behavior in donor–acceptor DCLs. Two of the acceptor building blocks differed in the structural features of the central core, as well as the length and hydrophilicity of the side chains. Screening of libraries containing different combinations of these donor and acceptor building blocks allowed us to investigate the extent to which all of these structural and electronic parameters play a role in the formation of distinct types of dynamic disulfide architectures.

### 2.2. Synthesis and Characterization of the New Thiol-Functionalized Viologen Component **7**

**Scheme 1:**
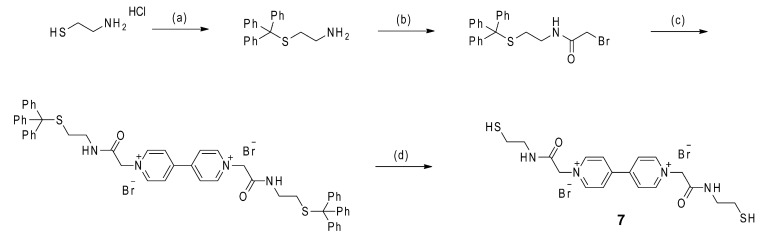
Synthesis of thiol-functionalized viologen Component **7**. (**a**) TFA, triphenylmethanol, room temperature, 3 h, (52%); (**b**) 2-Bromoacetic acid, *N*,*N′*-dicyclohexylcarbodiimide, CH_2_Cl_2_, room temperature, 24 h, (60%); (**c**) 4,4′-Bipyridine, MeCN, N_2_, 80 °C, 12 h (85%); and (**d**) TFA, Et_3_SiH, room temperature, 2 h (75%).

The synthesis of the thiol-functionalized viologen Component **7** is outlined in Scheme 1. A prerequisite to the generation of Structure **7** was the synthesis of *N*-(2-*S*-tritylethyl)-bromoacetamide. The latter molecule was synthesized according to the previously described procedure [7]. In the first step, protection of the thiol group of commercially available cysteamine hydrochloride was performed through a reaction with trifluoroacetic acid and triphenylmethanol to yield 2-tritylsulfanyl-ethylamine [43]. This was reacted with bromoacetic acid in dry DCM (Dichloromethane) at room temperature, using the peptide coupling agent *N*′*N*-dicyclohexylcarbodiimide as the carboxyl group activator to give the desired bromoacetamide molecule in a 60% yield. After reaction, the crude product was purified by column chromatography on silica gel with DCM as the eluent or was recrystallized from an acetone-water mixture. The thiol-protected viologen salt was obtained by nucleophilic substitution (S_N_2) of 4,4′-bipyridine using an excess of *N*-(2-*S*-tritylethyl)-bromoacetamide in acetonitrile (MeCN). The product was washed with acetonitrile and isolated as a bright yellow powder by suction filtration. Due to the highly hygroscopic character of the obtained material, the deprotection of the thiol group was performed directly after its isolation. Removal of the trityl group by using TFA and triethylsilane (TES) as a cation scavenger led to the desired Component **7** in the form of a yellow solid in a 75% yield. 

**Figure 2:**
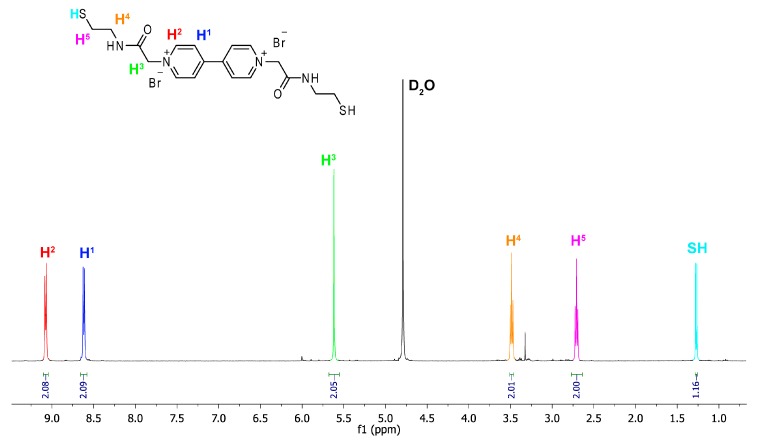
Part of the 500-MHz ^1^H NMR spectrum of thiol-functionalized viologen Component **7** in D_2_O.

The proton NMR spectra of Component **7** show signals expected for the symmetrically-functionalized viologen derivative (Figure 2). Of particular importance was the presence of a doublet at 1.26 ppm, which could be assigned to the thiol moiety in the reduced form. The latter factor is especially relevant when setting up multicomponent combinatorial libraries for the formation of disulfide architectures. Two triplets (H^4^ and H^5^) at 2.71 and 3.49 ppm and a singlet (H^3^) at 5.61 ppm represent –CH_2_– groups and are diagnostic for the successful substitution of the functional group, *N*-(2-*S*-tritylethyl)-bromoacetamide on both sides of the aromatic core. Finally, the two doublet signals in the aromatic region at 8.60 and 9.06 ppm (H^1^ and H^2^, respectively) can be assigned to the doubly-substituted viologen derivative.

### 2.3. Three-Component DCL

The first aqueous disulfide DCL was created by air oxidation of a 5 mM equimolar solution of Components **1**, **6** and **7** in water at pH 8. The pH was adjusted with aqueous NaOH, and the library was stirred and equilibrated under air in a closed-capped vial. After five days, oxidation was complete, and the library was then analyzed by reversed-phase HPLC (High-Performance Liquid Chromatography) and LC-MS (Liquid Chromatography-Mass Spectrometry). Absorbance measurements were made at 260 and 383 nm, the optimum wavelengths for the substituted naphthalene (donor) and naphthalenediimide (acceptor) components, respectively, and the library members were unambiguously identified by tandem ESI-MS (Electrospray Ionisation-Mass Spectrometry).

**Figure 3:**
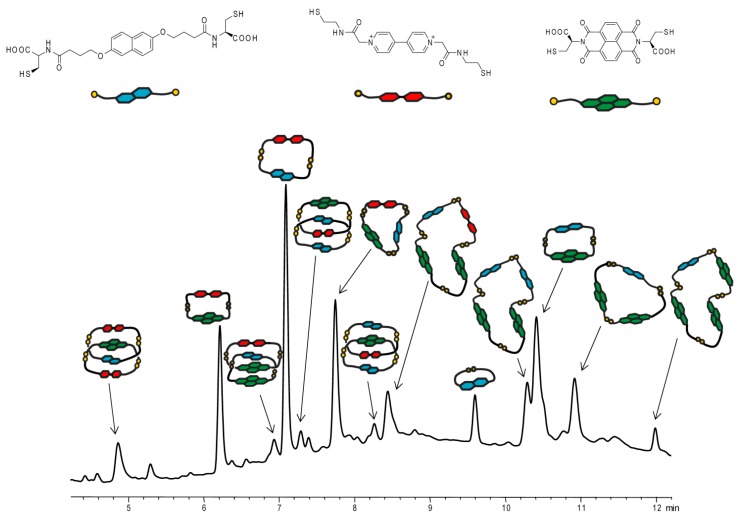
High-Performance Liquid Chromatography (HPLC) analysis of an aqueous library composed of **1**, **6** and **7** (1:1:1 molar ratio, 5 mM total).

At equilibrium, the three-component mixture provided thirteen structurally-distinct disulfide architectures together with several undefined peaks. The latter represented a small percentage of the library material (less than 5%), and in spite of attempts, they could not be identified so as to reflect the molecular weight of the components employed. The species containing only one kind of building block was the cyclic monomer consisting of **1** (retention time ≈ 9.6 min; Figure 3). Several macrocycles with different numbers of components that incorporate both donor and acceptor subunits were also present. Among them were three macrocycle species consisting of two distinct building blocks, out of which one had two acceptor Components **6** and **7** (retention time ≈ 6.3 min). Further analysis revealed the presence of two three-component and three four-component macrocyclic architectures. It is worth noting that two of them contained three different building blocks (retention times ≈ 7.8 and 8.5 min), which indicates good mixing between the structures employed.

Further analysis of this DCL indicated the presence of four [2]catenane architectures. These library members were present in insignificant amounts (less than 7% of the total library material) and differed in the composition of the macrocycles constituting [2]catenane systems. Four different D–A patterns in the [2]catenanes were observed. Three of them represent electronically-unfavorable architectures and have either three acceptor molecules and one donor or two donors and two acceptors in the configurations: AADA (retention time ≈ 4.9 min), ADAA (retention time ≈ 6.9 min) and DAAD (retention time ≈ 8.3 min). The remaining catenane had a conventional interlocked structure made up of two molecules of a donor and two acceptors in the ADAD pattern (retention time ≈ 7.3 min). Somewhat surprising is the low concentration of the favorable ADAD catenane, which may suggest that the electronic effect between donor and acceptor components is not the major driving force in the preferable formation of interlocked structures in the presented system. The results obtained indicate also that simultaneous formation of multicomponent and constitutionally-distinct [2]catenanes presenting different stacking patterns is readily accessible.

**Figure 4:**
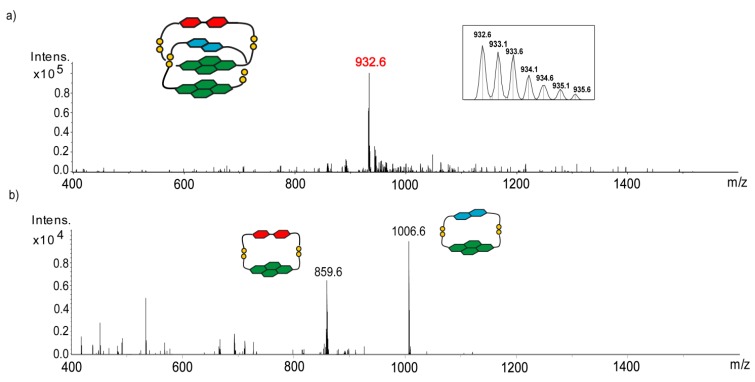
(**a**) ESI-MS and (**b**) MS/MS fragmentation of the ADAA (A, acceptor; D, donor; Intens., Intensity) catenane (molecular ion highlighted in red). A fragmentation amplitude of 0.6 V was used. The orange dots represent the sulfur atoms of the cysteine residues in the disulfide links.

The interlocked nature of [2]catenanes generated in the three-component DCL was unambiguously established by tandem MS (MS/MS) analysis. An illustrative example of ESI-MS and MS/MS analysis of ADAA [2]catenane (retention time ≈ 6.9 min) is shown in Figure 4. The ESI-MS spectrum (negative ion) shows a doubly-charged molecular ion (*m*/*z* of 932.6), corresponding to the mass of a tetramer composed of three donor (two of **6** and one of **7**) and one acceptor (**1**) units. The two major fragments observed in MS/MS have an *m*/*z* of 1006.6 and 859.6, corresponding to the mass of the macrocyclic heterodimers consisting of **1**–**6** and **6**–**7**, respectively. The direct fragmentation of a tetramer to two dimeric species is characteristic of an interlocked catenane structure.

### 2.4. Four-Component DCL

**Figure 5:**
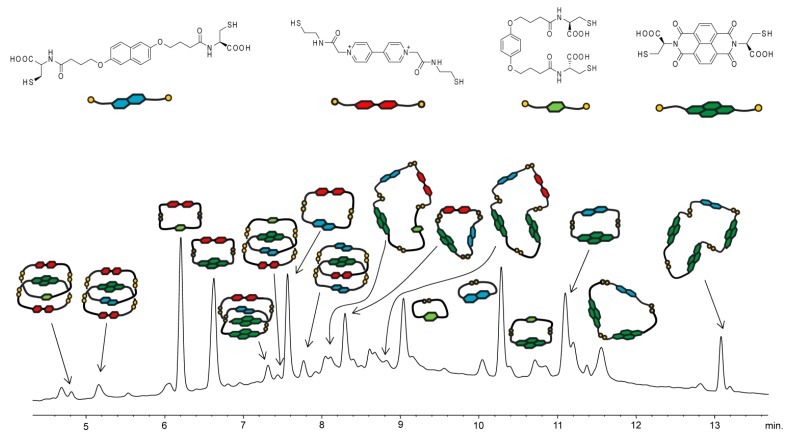
HPLC analysis of an aqueous library composed of **1**, **4**, **6** and **7** (1:1:1:1 molar ratio, 5 mM total).

To stimulate the emergence of a constitutionally more complex DCL containing a larger number of morphologically distinct D–A architectures, we decided to increase the number of dithiol building blocks employed in the experiment. Figure 5 shows the DCL consisting of four components, namely: **1**, **4**, **6** and **7**. In contrast to the previously described DCL, two structurally different donor (**1** and **4**) and acceptor (**6** and **7**) components were used in the formation of disulfide architectures. Once the system was observed to have reached equilibrium, seventeen different disulfide architectures were detected together with a number of unidentified species (less than 6% of the total library material; the observed molecular weight could not be assigned to any composition of the component employed). Five of these architectures had a [2]catenane topology; the rest were identified as macrocycles of different sizes (from monomeric to tetrameric). It is worth noting the emergence in the DCL of a [2]catenane consisting of four distinct components (retention time ≈ 7.5 min). This structure is present in a very small quantity, but represents an electronically-favorable architecture with two acceptor and two donor molecules in the configuration DADA (A, acceptor; D, donor).

To define the full spectrum of capabilities of the system, we introduced building blocks with minor structural modifications, namely Components **2** and **5**, which have shorter thiol linkers (only one –CH_2_– group between ether and carbonyl) compared to **1** and **4**. The DCL composed of **2**, **5**, **6** and **7** led to a significantly less complex library with eleven major disulfide structures present (see ESI). It is reasoned that this new constitutionally-simple library results from the inability of the shorter side chain in Components **2** and **5** forming some previously seen disulfide architectures, due to flexibility limitations. These results are in line with previous studies of donor–acceptor DCLs, where very detailed structural and mechanistic pathways were described showing a strong correlation between structural features of the thiol building block and the composition of the disulfide architectures observed [29].

In the last stage of our studies, Component **3** was introduced. This donor structure has a different substitution pattern of the functional thiol units on the central aromatic core (1,5- compared to 2,6- in **1** and **2**). A DCL obtained from this molecule together with **5**, **6** and **7**, similarly to the previously discussed case, showed a constitutionally-limited composition of disulfide products (see ESI). Twelve major disulfide architectures were observed along with some minor unidentified species. Interestingly, the major signal observed in the HPLC chromatogram appeared to be due to the [2]catenane in the configuration AADA (A, acceptor; D, donor). Its relatively short retention time (≈4.6 min) is due to the presence of two molecules of **7** (one in each macrocycle), which together with Components **3** and **6** form this architecture. This observation, together with the previously discussed results, suggests that by modifying the length and position of the functional side chain, we can, to a certain degree, favor the formation of certain disulfide architectures over others. Under the conditions of our experiments, it seems that the involvement of up to four thiol components leads to libraries that are either dominated by a large number of disulfide macrocyclic products (for components possessing long functional side chains and naphthalene substituted in the 2,6-position) or are relatively simple (for components possessing short functional side chains and/or naphthalene substituted in the 1,5-position) due to a significant part of the library material being consumed in the formation of [2]catenane architectures.

## 3. Experimental Section

### 3.1. Materials

Chemicals were purchased from commercial suppliers and used as received. Water and MeOH for LC-MS were purchased from Romil (Waterbeach, Cambridge, UK) or Rathburn (Walkerburn, Peeblesshire, UK).

### 3.2. HPLC and LC-MS

HPLC/LC-MS was performed on HP1050 (LabX, San Diego, CA, USA) or Agilent 1100LC/MSD trap XCT systems (Encino, California, CA, USA) coupled to a diode array detector, and the data were processed using ChemStation software. Mass spectra (negative mode) were acquired in ultra-scan mode using a drying temperature of 325 °C, a nebulizer pressure of 55 psi, a drying gas flow of 10 L/min, a capillary voltage of 4000 V, an ICC (Integrated Ion Current) target of 200,000 ions and a target mass of 1000. Analytical separations were achieved by injecting 5 µL (for 5 mM DCL, scaled accordingly for DCL at different concentrations) of DCL solution on to a Symmetry C8 reverse phase column (250 mm × 4.6 cm, 3-µm particle size, Waters, Milford, MA, USA) with an isocratic elution of 58% MeOH in water (with 0.1% formic acid) at room temperature and a flow rate of 1 mL/min.

### 3.3. Generation of DCL

A typical analytical DCL was prepared on a 0.8-mL scale by dissolving an equimolar mixture of dithiol components in 10 mM aqueous NaOH, followed by titration with 100 mM aqueous NaOH or HCl to pH = 8. The DCL was stirred in a close-capped vial at room temperature until being analyzed. The pH of each library was checked before and after the equilibration process to make sure it remained unchanged.

The choice of pH = 8 was dictated for the following reasons:

(1) a pH around 8 was found to be optimum for disulfide exchange rates;
(2) to ensure that the all of carboxylates are in their deprotonated form;
(3) the solubility of building blocks used is optimum at pH around 8.

### 3.4. Synthesis

The donor building Blocks **1**–**5** were synthesized following previously reported procedures [28,29,30,32,33,44,45]. They differ in the substitution pattern of the central core (1,5- or 2,6-) and the length of the side chains. Syntheses of the acceptor building Block **6** have already been published [23]. The detailed synthetic procedure for the new dithiol Component **7** and its protected version is outlined below.

#### Synthetic procedure for the *S*-trityl protected Molecule **7**:

*N*-(2-*S*-tritylethyl)-bromoacetamide (467.4 mg, 1.06 mmol) and 4,4′-bipyridine (66.3 mg, 0.42 mmol) were suspended in dry MeCN (20 mL). The reaction mixture was stirred under a N_2_ atmosphere at 80 °C for 12 h. The resulting yellow precipitate was isolated through suction filtration under N_2_ atmosphere and washed with dry MeCN (20 mL). Due to the highly hygroscopic character of the isolated yellow solid, the obtained material was used immediately in the deprotection step without further characterization.

#### Synthetic procedure for the unprotected Component **7**:

To the two-necked flask, *S*-trityl-protected Molecule **7** (200 mg, 0.21 mmol) was added under a N_2_ atmosphere. The flask was degassed three times, and after, TFA (1.5 mL, 27.8 mmol) was added. The resulting yellow solution was stirred under a N_2_ atmosphere at room temperature for 2 h. After this time, Et_3_SiH (80 µL, 1.28 mmol) was added dropwise via syringe, and stirring was continued for another 30 min. After this time, the solvent was removed, and to the yellow residue, Et_2_O (20 mL) was added. The yellow solid was filtered under a N_2_ atmosphere to give the product in a 75% yield.

^1^H NMR of **7** (500 MHz, D_2_O) δ 9.09 (d, *J* = 8.5 Hz, 2 H), 8.63 (d, *J* = 9.0 Hz, 2 H), 5.62 (s, 2 H), 3.49 (t, *J* = 8.0 Hz, 2 H), 2.71 (t, *J* = 8.0 Hz, 2 H), 1.28 (d, *J* = 8.0 Hz, 1 H).^13^C NMR (125.75 MHz, D_2_O) δ (ppm): 169.2, 151.3, 145.7, 123.4, 55.2, 39.9, 26.7. ESI-MS: observed [M]^2+^: 392.0265; calculated: 392.1374. Elemental analysis calcd. (%) for C_18_H_24_Br_2_N_4_O_2_S_2_: C 39.14, H 4.38, N 10.14; found: C 38.92, H 4.57, N 10.09.

## 4. Conclusions

In this work, we have extended the disulfide donor–acceptor DCC chemistry by introduction of a new type of thiol-functionalized building block. This viologen-based electron-deficient structure was found to be very effective in promoting the formation of a series of new D–A disulfide architectures, including [2]catenanes. The latter structure consists of four structurally-distinct components. We have also found that by manipulation of the structural features of the thiol components (changing the length or the position of the linker), the constitution of the DCL is modified. In libraries consisting of four thiol components (both acceptors and donors), the generation of up to seventeen disulfide structures was observed. These results led us to conclude that the right balance between flexibility and rigidity is an extremely important element in self-assembled dynamic systems.

## References

[1] Aleman Garcia M.A., Bampos N. (2013). Synthesis of a four-component [3]catenane using three distinct noncovalent interactions. Org. Biomol. Chem..

[2] Liu X.J., Warmuth R. (2006). Solvent effects in thermodynamically controlled multicomponent nanocage syntheses. J. Am. Chem. Soc..

[3] Sakai N., Lista M., Kel O., Sakurai S.-I., Emery D., Mareda J., Vauthey E., Matile S. (2011). Self-organizing surface-initiated polymerization: Facile access to complex functional systems. J. Am. Chem. Soc..

[4] Stefankiewicz A.R., Harrowfield J., Madalan A., Rissanen K., Sobolev A.N., Lehn J.M. (2011). Structural and metallo selectivity in the assembly of (2 × 2) grid-type metallosupramolecular species: Mechanisms and kinetic control. Dalton Trans..

[5] Stefankiewicz A.R., Rogez G., Harrowfield J., Drillon M., Lehn J.M. (2009). Structural features directing the specificity and functionality of metallo-supramolecular grid-type architectures. Dalton Trans..

[6] Stefankiewicz A.R., Rogez G., Harrowfield J., Sobolev A.N., Madalan A., Huuskonen J., Rissanen K., Lehn J.M. (2012). Self-ordering of metallogrid complexes via directed hydrogen-bonding. Dalton Trans..

[7] Dirk G.K. (2008). Metallo-supramolecular modules as a paradigm for materials science. Sci. Technol. Adv. Mater..

[8] Ciesielski A., Stefankiewicz A.R., Hanke F., Persson M., Lehn J.M., Samori P. (2011). Rigid dimers formed through strong interdigitated H-bonds yield compact 1D supramolecular helical polymers. Small.

[9] Herrmann A. (2014). Dynamic combinatorial/covalent chemistry: A tool to read, generate and modulate the bioactivity of compounds and compound mixtures. Chem. Soc. Rev..

[10] Jin Y., Wang Q., Taynton P., Zhang W. (2014). Dynamic covalent chemistry approaches toward macrocycles, molecular cages, and polymers. Acc. Chem. Res..

[11] Matache M., Bogdan E., Hădade N.D. (2014). Selective host molecules obtained by dynamic adaptive chemistry. Chem.-Eur. J..

[12] He Z., Jiang W., Schalley C.A. (2015). Integrative self-sorting: A versatile strategy for the construction of complex supramolecular architecture. Chem. Soc. Rev..

[13] Lehn J.M. (2007). From supramolecular chemistry towards constitutional dynamic chemistry and adaptive chemistry. Chem. Soc. Rev..

[14] Jin Y., Yu C., Denman R.J., Zhang W. (2013). Recent advances in dynamic covalent chemistry. Chem. Soc. Rev..

[15] Lehn J.-M. (2013). Perspectives in chemistry—Steps towards complex matter. Angew. Chem. Int. Ed..

[16] Lehn J.-M. (2015). Perspectives in chemistry—Aspects of adaptive chemistry and materials. Angew. Chem. Int. Ed..

[17] Mondal M., Hirsch A.K.H. (2015). Dynamic combinatorial chemistry: A tool to facilitate the identification of inhibitors for protein targets. Chem. Soc. Rev..

[18] Cougnon F.B., Sanders J.K. (2012). Evolution of dynamic combinatorial chemistry. Acc. Chem. Res..

[19] Pace G., Stefankiewicz A.R., Harrowfield J., Sarnori P., Lehn J.M. (2009). Self-assembly of alkoxy-substituted bis(hydrazone)-based organic ligands and of a metallosupramolecular grid on graphite. ChemPhysChem.

[20] Busseron E., Ruff Y., Moulin E., Giuseppone N. (2013). Supramolecular self-assemblies as functional nanomaterials. Nanoscale.

[21] Bulheller B.M., Pantos G.D., Sanders J.K.M., Hirst J.D. (2009). Electronic structure and circular dichroism spectroscopy of naphthalenediimide nanotubes. Phys. Chem. Chem. Phys..

[22] Whittell G.R., Hager M.D., Schubert U.S., Manners I. (2011). Functional soft materials from metallopolymers and metallosupramolecular polymers. Nat. Mater..

[23] Pengo P., Pantos G.D., Otto S., Sanders J.K.M. (2006). Efficient and mild microwave-assisted stepwise functionalization of naphthalenediimide with alpha-amino acids. J. Org. Chem..

[24] Waters M.L. (2013). Aromatic interactions. Acc. Chem. Res..

[25] Chung M.-K., Lee S.J., Waters M.L., Gagné M.R. (2012). Self-assembled multi-component catenanes: The effect of Multivalency and Cooperativity on Structure and Stability. J. Am. Chem. Soc..

[26] Chung M.-K., White P.S., Lee S.J., Waters M.L., Gagné M.R. (2012). Self-assembled multi-component Catenanes: Structural insights into an adaptable class of molecular receptors and [2]-catenanes. J. Am. Chem. Soc..

[27] Haino T., Araki H., Yamanaka Y., Fukazawa Y. (2001). Fullerene receptor based on calix[5]arene through metal-assisted self-assembly. Tetrahedron Lett..

[28] Au-Yeung H.Y., Cougnon F.B.L., Pantos G.D., Otto S., Sanders J.K.M. (2010). Exploiting donor-acceptor interactions in aqueous dynamic combinatorial libraries: Exploratory studies of simple systems. Chem. Sci..

[29] Cougnon F.B., Ponnuswamy N., Jenkins N.A., Pantos G.D., Sanders J.K. (2012). Structural parameters governing the dynamic combinatorial synthesis of catenanes in water. J. Am. Chem. Soc..

[30] Cougnon F.B.L., Au-Yeung H.Y., Pantos G.D., Sanders J.K.M. (2011). Exploring the formation pathways of donor-acceptor catenanes in aqueous dynamic combinatorial libraries. J. Am. Chem. Soc..

[31] Cougnon F.B., Jenkins N.A., Pantos G.D., Sanders J.K. (2012). Templated dynamic synthesis of a [3]catenane. Angew. Chem. Int. Ed..

[32] Au-Yeung H.Y., Pantos G.D., Sanders J.K.M. (2009). Amplifying different [2]catenanes in an aqueous donor-acceptor dynamic combinatorial library. J. Am. Chem. Soc..

[33] Au-Yeung H.Y., Pantos G.D., Sanders J.K.M. (2009). Dynamic combinatorial synthesis of a catenane based on donor-acceptor interactions in water. Proc. Natl. Acad. Sci. USA..

[34] Perez-Fernandez R., Pittelkow M., Belenguer A.M., Lane L.A., Robinson C.V., Sanders J.K.M. (2009). Two-phase dynamic combinatorial discovery of a spermine transporter. Chem. Commun..

[35] Han F.S., Higuchi M., Kurth D.G. (2007). Metallo-supramolecular polymers based on functionalized bis-terpyridines as novel electrochromic materials. Adv. Mater..

[36] Stefankiewicz A.R., Sanders J.K.M. (2013). Diverse topologies in dynamic combinatorial libraries from tri- and mono-thiols in water: Sensitivity to weak supramolecular interactions. Chem. Commun..

[37] Stefankiewicz A.R., Sambrook M.R., Sanders J.K.M. (2012). Template-directed synthesis of multi-component organic cages in water. Chem. Sci..

[38] Moulin E., Cormos G., Giuseppone N. (2012). Dynamic combinatorial chemistry as a tool for the design of functional materials and devices. Chem. Soc. Rev..

[39] Ladame S. (2008). Dynamic combinatorial chemistry: On the road to fulfilling the promise. Org. Biomol. Chem..

[40] Du D.-Y., Qin J.-S., Li S.-L., Su Z.-M., Lan Y.-Q. (2014). Recent advances in porous polyoxometalate-based metal-organic framework materials. Chem. Soc. Rev..

[41] Bhosale S.V., Jani C.H., Langford S.J. (2008). Chemistry of naphthalene diimides. Chem. Soc. Rev..

[42] Monk P.M.S. (1998). The Viologens: Physicochemical Properties, Synthesis, and Applications of the Salts of 4,4′-Bipyridine.

[43] Rao P.V., Bhaduri S., Jiang J., Holm R.H. (2004). Sulfur bridging interactions of cis-planar NiII-S2N2 coordination units with Nickel(II), Copper(I,II), Zinc(II), and Mercury(II): A library of bridging modes, including NiII(μ2-SR)2MI,II Rhombs. Inorg. Chem..

[44] Pantos G.D., Au-Yeung H.Y., Sanders J.K.M. (2011). Dynamic combinatorial donor-acceptor catenanes in water: Access to unconventional and unexpected structures. J. Org. Chem..

[45] Ho Yu A.-Y., Pantos G.D., Jeremy K.M.S. (2010). A water soluble donor-acceptor [2]catenane that can switch between a coplanar and a gemini-sign conformation. Angew. Chem. Int. Ed..

